# Incidence and anatomy of cardiac malformations in children conceived by assisted reproduction techniques - A review

**DOI:** 10.5935/1518-0557.20140005

**Published:** 2014

**Authors:** Paulo F. Taitson, Daniela D. Kwong, Gabriela Cristina de A. Lima, Ludmila S. Coelho, Wanessa D.Bruce, Nicole de O. Bernardes

**Affiliations:** 1 Discipline of Anatomy and Discipline of Human Reproduction, Pontifical Catholic University of Minas Gerais, Brazil; 2 Graduates from Pontifical Catholic University of Minas Gerais, Brazil; 3 Department of Phisioterapy, Pontifical Catholic University of Minas Gerais, Brazil

**Keywords:** Cardiac malformations, cardiac anatomy, infertility, human reproduction, health promotion

## Abstract

The aim of this study is to identify the occurrence of cardiac malformations occurred in children conceived by assisted reproduction techniques (ART) in the international literature in Medline database from 1999 to 2012.

The search returned data on 32,000 births, whose 21,000 were conceived naturally and 11,000 were conceived by ICSI and/or IVF. The incidence of cardiac malformations in general population situations was 0.4%.

The incidence of cardiac malformations by ART was 1.8% from ICSI and IVF. Among the situations of conceived naturally was observed 88 cases of cardiac malformations, which were: Atrial Septal Defect: 26 cases 29.55%, Change in the interventricular septum: 18 cases 20.45%, coarctation of the aorta: 13 cases 14.77%, aortic stenosis: 11 cases 12.5%, tetralogy of Fallot: 5.68% 5 cases, stenosis of the pulmonary trunk: 5 cases 5.68% other: 11.37% 10 cases. In cases of cardiac malformations in children conceived by IVF and ICSI, 198 cases were found, which were: CIA: 58 cases 29.30%, Change in the interventricular septum: 37 cases 18.69%, coarctation of the aorta: 20 cases 10.10%, aortic stenosis: 18 cases 9.09%, tetralogy of Fallot: 11 cases 5.55%, stenosis of the pulmonary trunk: 6 cases 3.03%, Other: 48 cases 24.24%. There remain opened questions about infertility and risk factors involving ART. Further research is clearly required. Health care providers should counsel infertile couples seeking assisted reproduction by IVF and technology.

## INTRODUCTION

Among the risks to female infertility are described ovulatory dysfunction, abnormal uterine tube, recurrent spontaneous abortion, pelvic endometriosis, genetic disorders and advanced age, among others.

The male reproductive potential status can result in more offspring in spite of increasing age. In fact, male and female lifetime strategies are as different as their mate selection criteria and the resulting cognitive differences. Azoospermia, the absence of spermatozoa in ejaculated semen, affects approximately 5% of all men and accounts for one-third of all male factor infertility cases (Nijs *et al*., 2011; Taitson *et al*., 2012).

Since the birth of the first infant conceived in the U.S. with assisted reproductive technology (ART) in 1981, the use of advanced technologies to overcome the problem of infertility has increased steadily. In 2009, (the most recent data available from the CDC) a total of 146,244 ART procedures resulted in 45,870 live-birth deliveries and 60,190 infants, or 1.4 % of U.S. births.

The proportion of conception assisted with in vitro fertilization (IVF) in some European countries is even higher. Among infants conceived with ART (in the U.S.), 33.4% were born preterm, compared with 12.2% of the general birth population, and 6.1% of ART infants were very preterm births, compared with 2% among the general birth population. The majority of this risk is due to the preterm delivery rates of multiple births. Among infants conceived with ART, 47% were born as multiple-birth infants compared with only 3% of infants among the general birth population. This represents a true iatrogenic health risk (Sunderam *et al*., 2012).

The many artificial procedures used during ART contribute to the concern that children conceived by ART might be exposed to greater health risks than naturally conceived (NC) children.

First, during the ART process, numerous medications are used to induce ovulation, gametes are recruited, embryos are cultured in an in vitro environment and then frozen and thawed, and large doses of progesterone are used to support the luteal phase. All these artificial procedures may harm the gametes and embryos. Furthermore, ICSI, which can fertilize an egg by directly injecting one sperm to the ooplasm, is more invasive than conventional IVF. ICSI also evades natural selection at the oocyte membrane and both genetically and structurally abnormal sperm will be able to fertilize eggs, which may pass abnormal genetic materials to the children. In addition, transferring more than one embryo significantly increases the rate of multiple pregnancies, which is associated with a higher rate of prematurity and low birth weights, carrying high risks of morbidity to the children (Alexander *et al*., 2005; Fauser *et al*., 2005).

A congenital heart defect is a problem with the structure of the heart. It is present at birth. Congenital heart defects are the most common type of birth defect. The defects can involve the walls of the heart, the valves of the heart, and the arteries and veins near the heart. They can disrupt the normal flow of blood through the heart. The blood flow can slow down, go in the wrong direction or to the wrong place, or be blocked completely. At least distinct types of congenital heart defects are recognized, with many additional anatomic variations.

Recent progress in diagnosis and treatment (surgery and heart catheterization) makes it possible to fix most defects, even those once thought to be hopeless: aortic valve stenosis (AVS), atrial septal defect (ASD), coarctation of the aorta (CoA), complete atrioventricular canal defect (CAVC), d-transposition of the great arteries, Ebstein’s anomaly, I-transposition of the great arteries, patent ductus arteriosis (PDA), pulmonary valve stenosis, single ventricle defects, tetralogy of Fallot, total anomalous pulmonary venous, truncus arteriosus defects, ventricular septal defect (VSD), (American Heart Association, 2013).

The aim of this study is to identify the occurrence of cardiac malformations occurred in children conceived by ART and revisit risk factors and preventive factors to the occurrence of cardiac malformations in the international literature.

## MATERIALS AND METHODS

Articles in the field of human reproduction published in the Medline database for the years 1999-2012 were searched. We considered the following key words: infertility and assisted reproduction techniques, cardiac malformations and techniques of human reproduction, Cardiac malformations and risk factors, and prevention Cardiac malformations. From 80 initially selected articles only 47 were used. The excluded items had no direct relationship with the research.

## RESULTS

There were reports of 32,000 births, 21,000 were conceived naturally conceived and 11,000 by ICSI or IVF. The incidence of cardiac malformations in natural situations was 0.4%.

The incidence of cardiac malformations in individuals conceived by ART was 1.8% from ICSI and/or IVF. Among the situations of normal fertilization were observed 88 cases of cardiac malformations: Atrial Septal Defect (ASD): 26 cases 29.55%, Change in the interventricular septum: 18 cases 20.45%, coarctation of the aorta: 13 cases 14.77%, aortic stenosis: 11 cases 12.5%, tetralogy of Fallot: 5.68% 5 cases, stenosis of the pulmonary trunk: 5 cases 5.68% Other: 11.37% 10 cases.

In cases of cardiac malformations in children conceived by ART (IVF and ICSI), 198 cases were found, which were: CIA: 58 cases 29.30%, Change in the interventricular septum: 37 cases 18.69%, coarctation of the aorta: 20 cases 10.10%, aortic stenosis: 18 cases 9.09%, tetralogy of Fallot: 11 cases 5.55%, stenosis of the pulmonary trunk: 6 cases 3.03%, Other: 48 cases 24.24%.

## DISCUSSION

Many studies have been performed concerning infants conceived by assisted reproductive techniques (ART). In the general population, 3% of surviving neonates have one major congenital anomaly at birth.

Some anomalies are detected during childhood or adolescence. The major cause of congenital anomalies is genetic factors which cause 50% of spontaneous abortions during the first trimester of pregnancy and 5% of neonatal deaths.

Some factors probably increase the incidence of congenital anomalies in ART infants. The reason for higher incidence of congenital anomalies in ART infants is due to the careful, continuous examination of these infants in comparison with normal infants. Some anomalies such as small umbilical hernias and pigmented skin spots or ear tags, which may not be reported in normal infants, are reported in ART infants (Nielsen & Wohlert, 1991; Sutcliffe, 2006).

Davies *et al.* (2012) in South Australia related the births and gestation period of at least 20 weeks or a birth weight of at least 400 g and registries of birth defects (including cerebral palsy and terminations for defects at any gestational period). They studied risks of birth defects (diagnosed before a child’s fifth birthday) among pregnancies in women who received treatment with assisted reproductive technology, spontaneous pregnancies (i.e., without assisted conception) in women who had a previous birth with assisted conception, pregnancies in women with a record of infertility but no treatment with ART and pregnancies in women with no record of infertility.

The increased risk of birth defects associated with IVF was no longer significant after adjustment for parental factors. The risk of birth defects associated with ICSI remained increased after multivariate adjustment, although the possibility of residual confounding could not be excluded (Davies *et al*., 2012).

The impact of congenital anomalies related to the techniques of ART depends on several factors, including its prevalence, the quality and availability of medical and surgical treatment, the risk factors and the effectiveness of primary prevention measures.

The knowledge of the most prevalent anomalies and possible risk factors may allow early intervention seeking primary prevention and positively impacting the quality of life of the child and family (Ooki, 2013).

Although the contributions of the techniques of ART, some issues have been discussed as potential risk suffered by children born from these procedures, such as congenital heart defects. The results show that the most incidents are: CIA, abnormal interventricular septum and coarctation of the aorta. Congenital malformations, coming from ART occur in accordance with a higher prevalence of certain risk factors, which may involve both the woman and the man.

The most prevalent risk factors are advanced age mothers, underlying causes of infertility and genetic factors, drugs used to induce ovulation or maintain pregnancy in the early stages of pregnancy and the frequent occurrence of multiple pregnancies (Hansen *et al*., 2002).

Despite all the benefits that the ART has brought to infertile couples, many studies are still needed so that we can establish a consensus about its harm both to couples and their children. One could say that the ART has had repercussions that shape a new reality to be evaluated in all its magnitude. There remain opened questions about infertility and risk factors involving ART. Further research is clearly required. Health care providers should counsel infertile couples seeking assisted reproduction by IVF and technology.
